# The Effect of Nighttime Rental Restrictions on E-Scooter Injuries at a Large Urban Tertiary Care Center

**DOI:** 10.3390/ijerph181910281

**Published:** 2021-09-29

**Authors:** Bjorn Anderson, Jonathan D. Rupp, Tim P. Moran, Lauren A. Hudak, Daniel T. Wu

**Affiliations:** 1 Emory University School of Medicine, Atlanta, GA 30303, USA; a.bjorn@wustl.edu; 2 Department of Emergency Medicine, Emory University School of Medicine, Atlanta, GA 30303, USA; tpmoran@emory.edu (T.P.M.); lhudak@emory.edu (L.A.H.); dtwu@emory.edu (D.T.W.); 3 Gangarosa Department of Environmental Health, Rollins School of Public Health, Emory University, Atlanta, GA 30303, USA; 4 Grady Memorial Hospital, Atlanta, GA 30303, USA

**Keywords:** micromobilty, nighttime ban, e-scooter injury costs

## Abstract

Safety policy for e-scooters in the United States tends to vary by municipality, and the effects of safety interventions have not been well studied. We reviewed medical records at a large, urban tertiary care and trauma center in Atlanta, Georgia with the goal of identifying trends in e-scooter injury and the effects of Atlanta’s nighttime ban on e-scooter rentals on injuries treated in the emergency department (ED). Records from all ED visits occurring between June 2018 through August 2020 were reviewed. To account for ambiguity in medical records, confidence levels of either “certain” or “possible” were assigned using a set of predefined criteria to categorize patient injuries as being associated with an e-scooter. A total of 380 patients categorized as having certain e-scooter related injuries were identified. The average age of these patients was 31 years old, 65% were male, 41% had head injuries, 20% of injuries were associated with the built environment, and approximately 20% were admitted to the hospital. Approximately 19% of patients with injuries associated with e-scooters noted to be clinically intoxicated or have a serum ethanol level exceeding 80 mg/dL. The implementation of a nighttime rental ban on e-scooter rentals reduced the proportion of patients with e-scooter injuries with times of arrival during the hours of the ban from 32% to 22%, however this effect was not significant (*p* = 0.16). More research is needed to understand how e-scooter use patterns are affected by the nighttime rental ban.

## 1. Introduction

Standing electric scooters (e-scooters) are a ubiquitous and increasingly utilized form of transportation. Although e-scooters may be user-owned, most users rent the devices through each operators’ cell phone applications with associated user agreements and safety training as well as use protocols accessed within the cellphone application. After completing user agreements, clients select their scooter, ride to their destination, and deposit the scooter without the need to locate a specific docking terminal. The affordability, accessibility, and ease of use have made these devices popular alternatives to traditional modes of transportation.

Early deployments of e-scooters often occurred without consultation with municipalities or, in some cases, as pilot deployments in collaboration with municipalities (e.g., Austin, TX and Portland, OR USA) [1,2]. Many of these municipalities had to rapidly develop policies governing e-scooter use that balanced a variety of concerns from the public with a rapidly growing e-scooter user base, the potential for e-scooters to provide last mile transportation in a sustainable mobility system, and the potential for e-scooters to increase access to transportation in underserved areas [1,2,3,4,5,6,7]. 

As e-scooter use has increased, injuries related to their use have become an important public health concern [5]. A retrospective analysis of data from the U.S. National Electronic Injury Surveillance System (NEISS) revealed a 354% increase in injuries from powered scooters in 18- to 34-year-olds between 2017 and 2018 (222% across all age groups), which coincides with the introduction of e-scooter ride-sharing programs in the United States [8]. Data from several retrospective series of emergency department (ED) cases indicate that injured riders are more likely to sustain extremity fractures and head injuries than other injury types and riders may be more likely to suffer fractures or head/face injuries while operating e-scooters than other forms of micromobility [9,10,11,12,13,14]. Consistent with these injury patterns and field observations, helmet use in the ED-treated population is low [13,14]. E-scooters injuries also represent a burden on the healthcare system, with between 80% and 90% of injured e-scooter riders requiring some form of radiologic imaging, and 14–33% requiring surgical intervention [9,10,12]

Despite this, surveillance of e-scooter injuries using medical data has been limited primarily because the injury coding system employed by hospitals, ICD-10, lacked coding specific to e-scooter and dockless electric micromobility device related injuries (henceforth referred to as e-scooter injuries) prior to October 2020 [14,15]. As a result, collection of medical data on e-scooter injuries has largely depended on key word searches of records and consistent and accurate medical documentation factors associated with injury such as helmet use and precrash behavior. This type of review is problematic partially because of ambiguity that exists in term “scooter”, which is colloquially used to describe e-scooters as well as mobility scooters (3-wheel powered wheelchairs), non-powered scooters, gas powered scooters, medical knee scooters, mopeds, and other devices. In addition, the wide variety in brand names combined with the variety of devices employed by individual brands compound the inconsistency in references to these devices in medical records [15].

As a relatively new form of mobility, the crash and injury rates of e-scooters have been compared to multiple other commonly used modes of transportation. A study in Austin, Texas utilizing vehicle miles traveled as reported by rental operators found a rate of injuries per vehicle miles traveled of 175 to 200 times the same injury rate for automobiles [16].

Since the introduction of e-Scooters in the U.S. in late 2017, multiple safety regulations and interventions have been implemented by municipalities to reduce e-scooter injuries [2,3,4,17]. In general, when regulations for e-scooter riders exist, riders are required to follow all traffic signs and signals and wear helmets if required by state or local ordinance [2,3,4]. Regulation addressing riding on sidewalks is mixed, with some municipalities (and some U.S. States) requiring on-road riding and others allowing riders on sidewalks [15]. Some of the most common safety interventions include improving bicycle lane infrastructure, sidewalk use restrictions, speed-governing, regulating device parking, and requiring operators to inform users of laws prior to initial device rental [2,12]. Nighttime restrictions on rentals have also been implemented [18]. Although data supporting the efficacy of some of these interventions exists in the cyclist literature, few of these interventions and policies have been evaluated to assess their potential to prevent e-scooter injuries [19,20,21,22,23,24].

In this study, we review medical records from a large, urban tertiary care and trauma center in Atlanta, Georgia to characterize trends in e-scooter injuries. The data collected are used to assess the effects of Atlanta’s nighttime ban on e-scooter rentals between the hours of 21:00 and 03:59 and characterize several factors that may modulate the risk of injury in electric standing scooter riders, including helmet use, alcohol intoxication, and the roadway/roadside environment. To the best of our knowledge this study represents that first analysis of the effects of a nighttime rental ban on e-scooter injury.

## 2. Materials and Methods

Medical Records from all emergency department visits to Grady Memorial Hospital (Atlanta, GA, USA) occurring between 1 June 2018 through 30 August 2020. Variables obtained included demographic information, means of arrival, time of arrival, insurance class, serum ethanol test results, provider notes, chief complaint, time of departure and departure disposition from the emergency department, acuity level, total charges, admission diagnosis codes, emergency department diagnosis codes, non-emergency department diagnosis codes, length of stay, and procedure codes. 

Provider notes from all patients treated during the study period were searched for the keywords related to use of e-scooters including: scooter, bird, byrd, lime, lyme, jump scooter, e-scooter, electric scooter, and “escooter”. These terms include the names of the companies operating e-Scooter fleets in the City of Atlanta during the study period as well as the terms “e-scooter” and “scooter” and common variations on the spelling of these terms. Records containing these keywords were then manually reviewed using the criteria in Table 1 and either identified as not being associated with an e-scooter or assigned a confidence level of either “certain” or “possible”. 

All medical records from patients with certain and possible e-scooter related injuries were then further reviewed and coded according to whether notes identified delays in presentation to the ED, mechanism of injury (fall, motor vehicle crash, struck by an object, struck against an object), helmet use (yes/no), whether the patient was intoxicated, and whether crashes were affected by the condition of the built-environment (i.e., injury was noted to have been affected by striking, riding over, or otherwise directly interacting with uneven/bumpy paved pathways, loose surface material such as rocks/gravel/dirt/sand/leaves/mud, train tracks, potholes/holes/cracks, and/or curbs). Records were also reviewed to identify complex presentations, which were defined as patients who had one or more unrelated complaints that increased the level of care required (e.g., concomitant syncope or acute psychiatric illness). Head injuries also identified by ICD-10 diagnosis codes S00–S09. 

Distributions of patient age, sex, alcohol intoxication (using an 80 mg/dL threshold), time of ED arrival (expressed as hour of the day in 24 h format), mechanism of injury, association of injury with condition of built infrastructure, helmet use, head injury, hospital charges, form of insurance, acuity level, and emergency department discharge disposition were calculated for e-scooter riders with injuries categorized as certain and possible. Categorical variables were described using frequencies and percentages. Age and charges were described using medians and interquartile ranges. 

Distributions of time of arrival were also compared prior to and following the implementation of a nighttime riding ban on 10 August 2019. Because time of day is cyclical, time of arrival was compared across e-scooter riders prior to and after the nighttime riding ban using the Watson-Wheeler test which evaluates whether two samples of circular data were drawn from the same population. 

Delayed presentations were excluded from all analyses involving time of ED arrival. A 4 h threshold was used to define a delayed presentation as descriptors of time of presentation-in provider note tended to be parts of the day (e.g., morning) rather than specific times. Analyses involving time of arrival were also repeated on the subset of patients who were transported to the emergency department via EMS, as the delay between the injury event and ED arrival is minimal in these cases. Delayed presentations were also excluded from analyses of alcohol use unless specific mention was made in the note that the patient endorsed alcohol use at the time of and preceding the injury inciting event. Complex presentations were also excluded from analysis of hospital charges, acuity level, and discharge disposition, as conditions unrelated to e-scooter injury could affected these variables. 

Across the entire dataset, less than 1% of the data were missing and the pattern of missingness was consistent with “missing completely at random” mechanisms (Little’s test *p* = 0.57). Missing data were multiply imputed using fully conditional specification [18]. Statistical analyses were conducted using R version 3.6.3 [19] 

## 3. Results

**Figure 1:**
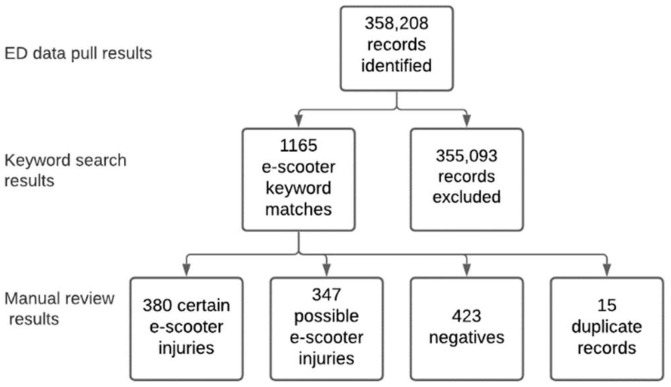
Results of the process used to identify e-Scooters from medical records.

As illustrated in Figure 1, of the ~360 k medical records examined during the study period, 380 patients with certain and 347 patients with possible e-scooter related injuries were identified. Within these groups, seven patients with certain e-scooter injuries and 25 patients with possible e-scooter injuries were identified as complex presentations. One-hundred seventy-nine patients with possible e-scooter injuries, and 227 patients with certain e-scooter injuries were transported by Emergency Medical Services (EMS).

Patients with injuries associated with e-scooters categorized as “certain” are described in Table 2. Key findings include that median age of these patients was 31 years old, 65% were male, 41% had head injuries, 20% of injuries were associated with the built environment, and approximately 19% were admitted to the hospital. Approximately 19% of patients with injuries associated with e-scooters noted to be clinically intoxicated or have a serum ethanol level exceeding 80 mg/dL.

Appendix A provides a description of the characteristics of patient categorized as “possible” and compares patients with injuries categorized as certain or possible. Results of this comparison show that patients categorized as possible e-scooter riders tended to be less severely injured (lower rates of hospital admission) than patients categorized as certain e-scooter riders. Consistent with less severe injury and the associated reduction in the number of provider notes, patients classified as possible e-scooter riders were less likely to be noted as clinically intoxicated and less likely to have the built environment noted as affecting crash causation. 

**Figure 2:**
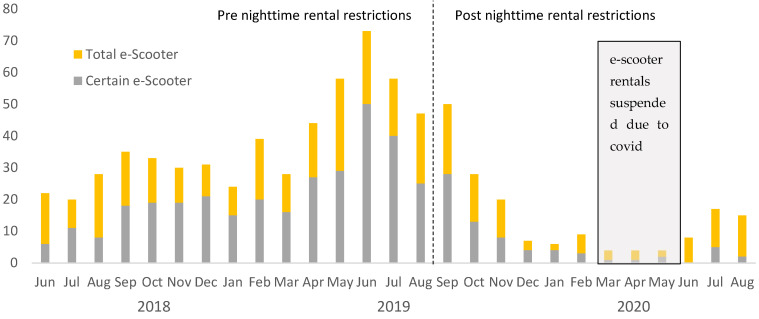
Total and certain e-scooter injuries by month.

Figure 2 shows trends in patients with e-scooter injuries by month. Consistent with trends in ridership reported to the City of Atlanta [25], the number of patients with e-scooter injuries peaked in summer 2019 and then decreased into the fall of 2019 and winter 2020 until the e-scooter rental program was briefly suspended due to the COVID pandemic. After this time, rentals and injuries associated with e-scooters resumed, albeit at a lower rate. Figure 2 also shows the expected seasonal variation in e-scooter injuries such that there is increased ridership in months where average temperatures are higher.

**Figure 3:**
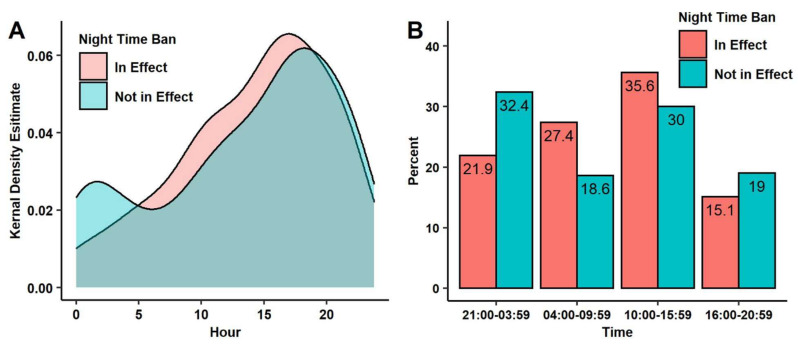

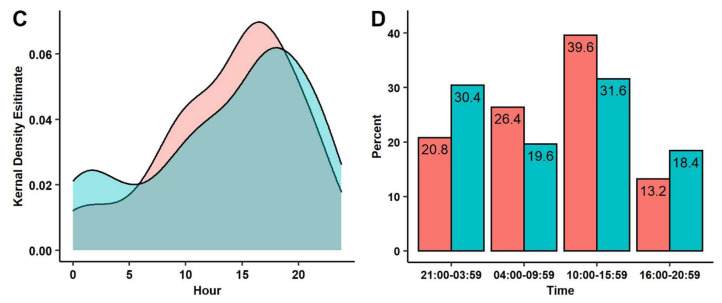
(**A**) A density plot depicting the distribution of arrival times for certain e-scooter patients as a function of the night time ban (12:00 a.m. is set to hour 0); the difference between the distributions was not significant (*p* = 23), (**B**) A bar graph depicting the percentage of certain scooter patients as a function of time of day and night time ban, the effect of the nighttime ban was not significant (*p* = 16), (**C**) A density plot depicting the distribution of arrival times for certain scooter patients transported by Emergency Medical Services (EMS) as a function of the night time ban; the difference between distributions is not significant (*p* = 0.18), (**D**) A bar graph depicting the percentage of certain scooter patients transported by EMS as a function of time of day and night time ban; the effect of the nighttime ban was not significant (*p* = 32).

As shown in Figure 3, for patients with certain e-scooter related injuries, the nighttime ban on rentals did not have a significant effect on ED time of arrival (*p* = 0.16), although the proportion of e-scooter riders injured during the hours of the night-time ban was reduced from 32% to 22% after the ban was instituted. This trend held for riders that were and were not transported by EMS as well as for analyses repeated using all certain and possible e-scooter riders not coded as having a delayed presentation to the ED.

## 4. Discussion 

Our results affirm that e-scooter injuries are a public health concern. Nearly 20% of injured e-scooter riders classified as certain in our sample had injuries severe enough to merit hospital admission. The median charges for care of these riders averaged $9.6 k. 

Approximately 16% of e-scooter riders classified as certain in our study were injured from interaction with motor-vehicles. This is slightly higher than the findings of previous studies where, motor-vehicles were associated with between 10% and 12.5% of injured patients [2,5,13,14]. Reasons for this difference unclear but may be associated with more and better infrastructure for cyclists in cities with lower rates of motor-vehicle involvement in e-scooter injuries.

Falls and the condition of the built environment were found to have an important role in the causation of injury for 73.9% and 19.7% of patients with e-scooter injuries classified as certain, respectively. Where documented, factors in the built environment associated with e-scooter injuries tended to be minor issues that are easier and faster to fix, such as debris, cracks, or potholes in paved roadways. Interaction with streetcar tracks was also noted as a problem. This finding is consistent with previous work in Austin, TX in which a study cosponsored by the CDC found that 50% of interviewed injured e-scooter riders considered poor condition of the built infrastructure contributed to their injuries [5]. Policy targeted appropriately to these issues may rapidly improve safe public use of e-scooters.

Injured riders of e-scooters were frequently diagnosed with head injuries. The observed rate of head injuries for injured e-scooter riders classified as certain was 40.8%. This value is within the range of previously published data, with literature revealing head injury rates between 10–48% [2,3,4,6,14,20]. Though the method of identification of head injuries in this study included less serious soft tissue injuries of the face and scalp, analyses unsurprisingly indicate that helmet use is associated with a decreased percentage of patients with head injuries. Despite the bias toward more severe injuries occurring from the use of an emergency department dataset, it is still likely that policies aimed at increasing helmet usage would reduce head injury rates in e-scooter riders.

Like other previous studies of e-scooter related injuries [2,5,13], our study identified few pedestrians injured as a result of being struck by e-scooters. This affirms the previous finding that, while a substantial proportion of complaints about e-scooter riders are related to sidewalk riding [5], interactions with e-scooter riders are not frequently injuring pedestrians severely enough to merit acute care.

The implementation of a nighttime rental ban on e-scooter rentals reduced the proportion of patients with e-scooter injuries who were transported by EMS with times of arrival during the hours of the ban, however this effect was not significant. Due to previous findings indicating that bicycle injuries are more frequent overnight and previous e-scooter fatalities in Atlanta occurring at night [23], it was expected that a nighttime rental ban would decrease the proportion of patients with e-scooter injuries arriving in the ED during the hours of the ban. The lack of a significant effect of the nighttime ban in our study may be from e-scooter-injuries occurring from rentals that began during allowable use hours and continued into the hours of the ban. 

Atlanta’s nighttime ban on rentals was intended to prevent more severe injuries primarily resulting from e-scooter crashes involving motor vehicles. Due to a relatively small post-ban sample size, the current study is not able to reliability estimate the effect of the nighttime ban on motor vehicle crashes involving e-scooters. The nighttime ban also ends at 04:00 h, meaning that depending on the time of year, it still allows substantial riding in predawn hours where visibility remains an issue.

Over the period of this study, the designs of e-scooters improved in ways that may have reduced crash rates and improved rider safety (e.g., larger tires and lower decks). A 24 km/h speed limit for e-scooters was also imposed. The combination of these factors likely influenced injury rates. However, as most of these improvements were implemented around the time of Atlanta’s nighttime rental ban they would have tended to exaggerate the effect of the ban on injury and there do not affect the finding that the ban did not significantly decrease the proportion of injured riders treated in the Emergency Department during the hours of the ban. 

The observed rates of injured e-scooter riders classified as certain with serum ethanol levels exceeding 80 mg/dL (9.1% certain) and serum ethanol combined with clinical assessment indicating intoxication (19.4% certain) are consistent with published intoxication rates for injured e-scooter riders treated in EDs in the United States, which range from 5.2–16% [9,12,14]. The reason for higher rates of alcohol impairment in our sample rate is unclear, however, e-scooter docking locations placed near destinations where alcohol is consumed and sold may play a role. It is also possible that impaired riders may be less likely to wear helmets. These data support the importance of efforts to reduce e-scooter use while intoxicated in reducing injury.

From February 2019 through August 2020, 296 patients categorized as certain and 586 total patients with injuries associated with e-scooter were identified. During the same time period, e-scooter riders reported 237 injuries to e-scooter rental companies that were subsequently reported to the City of Atlanta [21]. The lower numbers of injuries reported to rental companies is unsurprising and suggests that independent surveillance of e-scooter safety and injuries is important. Should independent surveillance be implemented, it should capture both emergency departments and urgent care locations, including those on college campuses, where e-scooters have been preferentially deployed in the U.S.

Despite the suspension of e-scooter rentals in Atlanta between late March and June 2020 due to coronavirus, 3 certain and 19 total e-scooter injured riders were identified in this study. These certain e-scooter injured riders may have resulted from either riding e-scooters without renting, or from privately owned devices of the same or similar design to those offered by ride-sharing services. 

## 5. Limitations and Future Work

As a retrospective single hospital medical chart review, this study has several limitations. First and foremost, these data are limited by the keyword search used to identify cases, which almost certainly did not capture all cases of e-scooter injury and likely categorized some patients whose injuries did involve e-scooters as being “possibly” injured involving an e-scooter. The keyword search was further complicated by conflicting terminology used between notes for the same patient and encounter. Furthermore, there are an unknown number of injuries resulting from e-scooter use that are not severe enough to prompt riders to present to emergency departments. The data from this study are therefore indicative of important trends, but generalization of the data is limited to injuries severe enough to prompt ED presentation. 

As ICD-10 coding was recently introduced allowing for more specific identification of e-scooter-related injury, further investigation of trends and factors of e-scooter injuries will become easier. However, full adoption and strict usage of these codes to identify all e-scooter-related injuries is unlikely to be immediate or complete. Though further study and data collection is important, the newness of e-scooters as an injury cause and heterogeneity in e-scooter types and terminology underscores the importance of manual review in continued research. The introduction of new ICD-10 coding more specific to e-scooters may improve this, but further research will be needed to evaluate the completeness and consistency of adoption of the new codes with regard to e-scooter injuries.

Our study focused on injuries involving e-scooters in an urban environment that prohibits sidewalk riding and that does not require helmet use. Our findings therefore may not be applicable to areas where riding on roadways is prohibited, where helmet use is required, or where riding is restricted to private property.

Our study was also unable to identify novice riders, who have been substantially overrepresented in among the e-scooter injured population in past studies [1,13]. We were also unable to characterize the effects of the COVID-19 pandemic on e-scooter injuries beyond that decreases in e-scooter trips during the pandemic reported to the City of Atlanta [25], Georgia reduced the number of injured riders treated in the ED. Future work should further explore how the pandemic has affected e-scooter ridership and injury taking into consideration pandemic related changes in ridership and rider behavior.

## 6. Conclusions

We performed a retrospective review of medical records from a single trauma center in Atlanta, Georgia, USA to characterize factors influencing e-scooter injury and study the effects of regulation banning e-scooter rentals between the hours of 21:00 and 03:59. Results from our analysis indicate that the nighttime ban decreased the proportion of e-scooter riders treated in the emergency department during the hour of the ban, but that the effect was not significant. Other findings with implications on future injury prevention interventions include that alcohol impairment was noted in 19% of injured e-scooter riders, that over 40% of injured riders had head/face injuries, and that the condition of the built environment was noted to be a factor for 20% of injured riders. 

## Figures and Tables

**Table 1:** Criteria Used to Identify and Assign Confidence to Patients with e-Scooters Injuries in Manual Review of Provider Notes.

**Certain**
Injury related to use of a e-scooter described as a motorized “upright”, “standing” or “push” scooter; “electric” or “electronic” “scooter”; “escooter” or “e-scooter”; and/or a specific brand of commercially available rentable e-scooter anywhere in documentation.
Mention of being “driver” of scooter described as “push”, “standing”, “stand up”, or “upright” in the absence of characterization as motorized or electric device anywhere in documentation.
**Possible**
Description of e-scooter as a scooter or motorized scooter without further characterization or along with use of “seated”, “seat,” “moped”, “motorcycle” or “bike” in description of device.
Criteria for certain not met.
**Negative**
Use of e-scooter at speeds unattainable by commercially available e-scooters and in described as being in a seated position/on a seat.
Mention of use or brand specific to non-e-scooters such as “vespa”, “kickstarting”, “switching gears”, “razor” or “arm rests”
Description of device as a mobility scooter
Patient has chronic medical condition prohibiting ability to use standing/upright electric scooter.
Description of the scooter as a wheelchair or dirt bike without meeting definite positive or possible criteria.
Presentation with psychiatric or medical chief complaint without evidence of trauma meeting definite positive or possible criteria.
Initial Patient presentation or EMS transport from a location outside deployment regions of commercially available rentable upright dockless electric scooters.
Subsequent encounters for the same e-scooter injury, or reinjury of old e-scooter-related injury by mechanism other than e-scooter use.
Criteria for certain and possible not met.

**Table 2:** Characteristics of Patients with Injuries with e-Scooters Injuries Classified as Certain.

**Characteristic**	**E-Scooter Median (IQR)**
Age (Years)	31 (23–41)
Charges (USD)	9608 (3591–24,252)
**Characteristic**	**N (%)**
Race/Ethnicity	
Native American	2 (0.5)
Asian	15 (3.9)
Black	211 (55.5)
Hispanic	18 (4.7)
Multi-Racial	8 (2.1)
Other	1 (0.3)
White	125 (32.9)
Time of Arrival ^†^	
21:00–03:59	96 (30.0)
04:00–09:59	41 (12.8)
10:00–15:59	80 (25.0)
16:00–20:59	103 (32.2)
Disposition ^††^	
Admitted	71 (19.0)
Discharged	299 (80.2)
Death	1 (0.3)
Transferred	2 (0.5)
Insurance	
Commercial	159 (41.8)
Medicaid	47 (12.4)
Medicare	14 (3.7)
Self-pay	160 (42.1)
Mechanism of Injury	
Fall	281 (73.9)
MV	61 (16.1)
Other/Unknown	10 (2.6)
Struck Against	23 (6.1)
Struck By	5 (1.3)
EtOH (serum ethanol)	
EtOH < 80 mg/dL	53 (16.6)
EtOH ≥ 80 mg/dL	29 (9.1)
Clinically Intoxicated	33 (10.3)
Not Intoxicated/Tested	205 (64.1)
Helmet	
Not Recorded	290 (76.3)
No	79 (20.8)
Yes	11 (2.9)
Built Environment	75 (19.7)
Head Injury	156 (41.1)

^†^ Excludes patients with delayed presentations; ^††^ Excludes patients with complex presentations.

## Data Availability

Data are stored in de-identified state and can be made available through a data use agreement.

## Appendix A

**Table A1:** Results of univariate statistical analysis comparing certain with possible bicycle injuries.

Characteristic	Certain Median (IQR)	Uncertain Median (IQR)	*p*	*p* (FDRa) ^†^
Age	31 (23–41)	31 (24–42)	0.55	1
Charges	9608 (3591–24,252.7)	6220.6 (2439.6–19,660)	0.004	0.03
**Characteristic**	**N (%)**	**N (%)**	***p***	
Race/Ethnicity			<0.001	<0.001
Native American	2 (0.5)	1 (0.3)		
Asian	15 (3.9)	5 (1.4)		
Black	211 (55.5)	254 (73.2)		
Hispanic	18 (4.7)	11 (3.2)		
Multi-Racial	8 (2.1)	2 (0.6)		
Other	1 (0.3)	3 (0.9)		
White	125 (32.9)	71 (20.5)		
Time of Arrival			0.57	1
Disposition			0.67	1
Admitted	71 (19)	50 (15.5)		
Discharged	299 (80.2)	269 (83.5)		
Death	1 (0.3)	1 (0.3)		
Transferred	2 (0.5)	2 (0.6)		
Insurance			0.002	0.02
Commercial	159 (41.8)	98 (28.2)		
Medicaid	47 (12.4)	57 (16.4)		
Medicare	14 (3.7)	12 (3.5)		
Self-pay	160 (42.1)	180 (51.9)		
Mechanism of Injury			<0.001	<0.001
Fall	281 (73.9)	233 (67.1)		
MV	61 (16.1)	54 (15.6)		
Other/Unknown	10 (2.6)	39 (11.2)		
Struck Against	23 (6.1)	9 (2.6)		
Struck By	5 (1.3)	12 (3.5)		
EtOH (serum ethanol)			0.11	0.82
EtOH <80 mg/dL	53 (16.6)	49 (20.7)		
EtOH ≥ 80 mg/dL	29 (9.1)	20 (8.4)		
Clinically Intoxicated	33 (10.3)	12 (5.1)		
Not Clinically Intoxicated/Tested	205 (64.1)	156 (65.8)		
Helmet			<0.001	<0.001
Not Recorded	290 (76.3)	265 (76.4)		
No	79 (20.8)	47 (13.5)		
Yes	11 (2.9)	35 (10.1)		
Built Environment	75 (19.7)	30 (8.6)	<0.001	<0.001
Head Injury	156 (41.1)	97 (28)	<0.001	0.003
Acuity			<0.001	<0.001
Emergent	30 (8)	25 (7.7)		
Immediate	10 (2.7)	12 (3.7)		
Less Urgent	60 (16.1)	94 (29.1)		
Non-Urgent	1 (0.3)	13 (4)		
Urgent	272 (72.9)	179 (55.4)		

^†^ False Discovery Rate corrected for multiple comparisons.
